# Intracranial germ cell tumors: advancement in genomic diagnostics and the need for novel therapeutics

**DOI:** 10.3389/fonc.2025.1513258

**Published:** 2025-01-31

**Authors:** Kee Kiat Yeo, Joanna Gell, Girish Dhall, Ching Lau

**Affiliations:** ^1^ Department of Pediatric Oncology, Dana-Farber/Boston Children’s Cancer and Blood Disorders Center, Boston, MA, United States; ^2^ Department of Pediatrics, Harvard Medical School, Boston, MA, United States; ^3^ Center for Cancer and Blood Disorders, Connecticut Children’s Medical Center, Hartford, CT, United States; ^4^ The Jackson Laboratory for Genomic Medicine, Framingham, CT, United States; ^5^ Department of Pediatrics, University of Connecticut School of Medicine, Framington, CT, United States; ^6^ Alabama Center for Childhood Cancer and Blood Disorders at Children’s of Alabama, Birmingham, AL, United States; ^7^ Department of Pediatrics, Marnix E. Heersink School of Medicine, University of Alabama at Birmingham, Birmingham, AL, United States

**Keywords:** germ cell tumor, central nervous system, intracranial, genomics, liquid biopsy, therapeutics

## Abstract

**Introduction:**

The outcomes for patients with intracranial germ cell tumors (GCT) has improved over the past few decades. However, there remains a lack of a consensus on a standard diagnostic and treatment approach of these tumors. The diagnostic work-up of intracranial GCT remains variable, and the treatment for patients with recurrent disease remains challenging.

**Methods:**

We review the current approach in the diagnosis and treatment of intracranial GCT. Given the heterogeneity of these tumors, we highlight the challenges and controversy with these conventional approaches.

**Results:**

We discuss the advancements in the understanding of the underlying genetic changes in intracranial GCT and the utility of novel molecular techniques in the diagnosis and classification of intracranial germ cell tumors as well as development of potential novel therapeutics.

**Discussion:**

Development of liquid biopsy platforms for diagnosis and management of malignancies is a rapidly growing field. Current approach utilizing traditional tumor markers have significant limitations. In this review, we will discuss profiling of intracranial GCTs for genetic and epigenetic signatures, which are emerging as promising biomarkers to assist in the diagnosis and management of intracranial GCTs. Various studies have shown that activating mutations in MAPK pathway are common alterations in intracranial GCTs, with KIT expression seen in most germinomas. Development of targeted therapeutics against KIT has led to the prospect of targeted therapy in germinoma. Other treatment modalities being considered for clinical development include immunotherapy and the use of immune checkpoint inhibitors, especially in NGGCT. In this review, we will discuss the potential novel therapeutics and the clinical trials that are currently under development.

## Introduction

Intracranial germ cell tumors (GCT) are a rare group of malignant tumors, most commonly arising in the second decade of life (1). Intracranial GCTs share histological, diagnostic, and therapeutic similarities with non-central nervous system (CNS) GCT, owing to their common cell of origin (2, 3). In the United States, intracranial GCTs represent 3-5% of all primary CNS tumors in pediatrics. The incidence is higher in East Asian countries such as Japan, with reported incidence of over 10%. As a whole, intracranial GCTs are significantly more common in males.

Intracranial GCTs are clinically divided into germinoma and non-germinomatous germ cell tumors (NGGCT). Germinomas are more common, accounting for approximately 2/3 of all intracranial GCTs. NGGCT are a heterogeneous group of tumors, including endodermal sinus (yolk sac) tumor, choriocarcinoma, embryonal carcinoma, teratoma (mature and immature) and mixed GCT (which can include components of germinoma). Intracranial GCTs most commonly arise in the midline structures of the CNS, primarily in the pineal and suprasellar regions (4). Rarely, these tumors can originate in other locations such as basal ganglia/thalamus, ventricles, and cerebral/cerebellar cortex.

Over the past few decades, clinical outcomes for patients with intracranial GCTs have improved, in part through collaborative clinical trials that have evaluated various diagnostic and therapeutic regimens. Despite these successes, there remains a lack of a universally accepted consensus on the diagnostic work-up and management for these tumors. In this review, we discuss the advancement in molecular genetics, the development of and the potential utility of innovative techniques in the diagnosis of intracranial GCT, as well as several novel therapeutic strategies that are currently being considered for clinical trial development for these tumors.

## Diagnosis

### Current approach

At present, the clinical diagnosis of intracranial GCTs relies on a combination of imaging characteristics and the presence of tumor markers, namely alpha-fetoprotein (AFP) and beta subunit of human chorionic gonadotropin (β-HCG), in the serum and/or cerebrospinal fluid (CSF). For cases where tumor markers are negative, surgical biopsy is recommended for histopathological confirmation (5). In addition to the characteristic morphological appearance on histology, common immunohistochemical (IHC) analysis used for the diagnostic work-up for GCT include CD117/KIT (germinoma), POU5F1/OCT4 (germinoma), Placental alkaline phosphate (PLAP) (germinoma), AFP (yolk sac tumor), CD30 (embryonal carcinoma), and HCG (choriocarcinoma or syncytiotrophoblast in germinoma).

Although these measures have been the standard of diagnostics for decades, they are imperfect. For instance, conventional tumor markers have low sensitivity and specificity, with some studies reporting only one-third of patients with intracranial GCT being tumor marker positive (6, 7). This low frequency is in part related to the predominance of germinomas within intracranial GCTs, where the majority of germinomas do not secrete tumor markers. For the minority of germinomas that do secrete β-HCG, they generally have low-level marker elevation and is likely related to the presence of syncytiotrophoblastic elements. Importantly, while β-HCG secreting germinomas is a widely accepted entity, there remains a lack of consensus on the cut-off level of β-HCG for the diagnosis of germinoma versus NGGCT.

For example, in Children’s Oncology Group (COG) trials, β-HCG cut-offs of up to ≤ 100 IU/L have been used to indicate pure germinoma histology, however, European SIOP trials have used a more conservative cut-off of ≤ 50 IU/L (8, 9). In Japan, a histopathologic-based diagnosis is generally preferred and used in their clinical trials, except for extreme instances such as β-HCG levels of >2,000 IU/L, which would indicate NGGCT, such as choriocarcinoma (10). Similarly, there are different consensus cut-off for AFP levels. AFP > 10ng/ml (or > upper limit of normal) is used in COG trials, whereas in European trials a level >25 ng/ml has been used as an indicator of a NGGCT (8, 9). Like germinomas, teratomas are often tumor marker negative; while this holds true for pure mature teratomas (MT), immature teratomas (IT) may secrete AFP. The AFP level that indicates an IT has not been well established, with examples of extracranial pure ITs having mean AFP levels of approximately 30-80 ng/ml (11).

Importantly, even in instances where histopathologic diagnosis is obtained through tissue biopsy, sampling error remains a significant concern. This is particularly challenging, especially with the known predilection of these tumors to have mixed histology. For instance, a marker negative tumor is often a mixed tumor containing numerous distinct components. However, a biopsy may capture only the germinoma component, leading to inadequate treatment. This is of critical clinical significance, as the treatment regimens for germinoma and NGGCT vary significantly, and with differing survival outcomes. Finally, other non-Intracranial GCT entities can mimic marker negative intracranial GCTs, such as Langerhans Cell Histiocytosis (LCH) and lymphocytic hypophysitis. Given these imperfect means of diagnosing these tumors, efforts to enhance accuracy of diagnosis, identify potential biomarkers that are predictive and prognostic, are imperative.

## Treatment

### Current approach

Despite considerable variation in treatment regimens commonly used in North America, Europe, and Asia, the general strategy for intracranial GCT involves surgery for diagnosis and/or CSF diversion, and the combination of chemotherapy and radiation therapy.

#### Germinoma

Through a series of clinical trials (International CNS Germ Cell Tumor Studies), chemotherapy-alone approaches were previously shown to be insufficient for the treatment of germinoma. Chemotherapy-alone approaches were associated with a temporary response with high rates of recurrence, resulting in a cure rate of less than 50% (12, 13). In contrast, high-dose craniospinal irradiation (CSI) alone has been shown to achieve durable remission and high rates of cures in germinomas, regardless of metastatic status (14).

In the last few decades, the focus of many clinical trials has revolved around reduction of radiation therapy and minimizing long-term treatment-related toxicity. As a result, neoadjuvant chemotherapy has been incorporated into the treatment regimens for germinoma prior to radiation therapy, an approach which has been successful in reducing the dose and/or field of radiation therapy needed to maintain the excellent cure rates (15, 16).

#### Non-germinomatous germ cell tumor

In contrast, NGGCTs are relatively more resistant to treatment and associated with a poorer prognosis. Previous efforts to evaluate treatment with either chemotherapy-only or CSI-only approaches were similarly inadequate, with unacceptably high rates of disease recurrences (12, 17–19). It is now clear that the combination of chemotherapy followed by radiation therapy is essential to improving outcome for these patients (8, 9, 20). The optimal chemotherapy regimen and radiation therapy plan, however, remains unclear (21). This is especially true for patients with localized disease, where the optimal radiation therapy plan remains undetermined. The current COG trial, ACNS2021, aims to determine if the addition of spinal canal irradiation to whole ventricular irradiation (after induction chemotherapy), will decrease the number of spinal relapses that was seen in prior studies (NCT04684368).

#### Recurrent intracranial GCT

Despite overall improving outcomes with combinatorial therapy approaches, a proportion of patients with intracranial GCT suffer from relapse or refractory disease. Treatment options for these patients are less unified and curative options are more limited. For those with recurrent intracranial germinoma, they are more likely to respond to additional chemotherapy and achieve durable remission with re-irradiation therapy (22). In contrast, those with recurrent or refractory NGGCT have more aggressive disease and significantly worse outcomes. Several chemotherapy regimens have been evaluated as salvage therapy for these patients, with variable response. Most recently, a phase 2 trial of GemPOx (Gemcitabine, Paclitaxel, Oxaliplatin) demonstrated that this combination was an active salvage therapy, effective in facilitating stem cell mobilization and enabling high-dose chemotherapy with autologous stem cell rescue as well as re-irradiation therapy in a significant proportion of patients (23). However, despite initial responses, majority of patients ultimately died from recurrent/refractory disease. The result of this trial is similar to other publications that show that despite aggressive salvage therapies, prognosis of recurrent/refractory intracranial GCT remains poor, and novel therapeutic approaches for these patients are needed (24, 25).

## Discussion

### Emerging technologies and biomarkers

In recent years, there have been substantial advancement in the understanding of the molecular basis of intracranial GCTs. However, due to the rarity of these tumors and lack of adequate tissue samples, molecular profiling has been challenging. In recent years, utilizing blood and/or CSF as an alternative has been evaluated by various groups. Given that CSF collection is a standard component of the diagnostic workup and evaluation of response to therapy, several groups have sought to evaluate CSF for novel biomarkers of intracranial GCTs.

One such example is with MicroRNAs (miRNAs), which has been emerging as a novel biomarker for several malignancies, including GCTs. MiRNAs have been studied extensively in extracranial GCTs, particularly in testicular GCTs (26–28). In extracranial GCTs, the miRNA clusters (miR-371-373 and miR-302/367) have been identified as biomarkers of malignant GCTs, but are notably not expressed in benign teratomas (26, 29). Specifically, miR-371a-3p has emerged as a highly sensitive and specific marker of malignant testicular GCTs (30). In patients with intracranial GCTs, two small case series have recently demonstrated the feasibility of detecting these two miRNA clusters (31, 32). Despite promise, larger validation studies will be needed to demonstrate reproducibility of this methodology, as well as evaluate the sensitivity and specificity of these miRNA clusters in the setting of intracranial GCTs. If miRNAs prove to be a sensitive diagnostic tool for detecting intracranial GCTs, this could potentially be beneficial to the group of patients who present with neuroendocrine dysfunction and slowly growing suprasellar lesions, who often have a delay in diagnosis due to negative tumor markers and insufficient mass for biopsy (33, 34).

In addition to miRNA, circulating tumor DNA (ctDNA) is another evolving field in oncology that holds immense promise. Particularly in CNS tumors, various researchers have looked at the utility of CSF to identify recurrent molecular alteration, both at diagnosis and for disease monitoring (35–37). Recurrent somatic mutations in the KIT/RAS/MAPK pathways and AKT/mTOR pathways have been well documented in intracranial GCTs, with KIT/KRAS/MAPK alterations known to be enriched in germinomas. Takayasu et al., analyzed 8 germinomas and 4 NGGCTs for the presence of ctDNA in CSF of patients, utilizing a next generation sequencing (NGS) panel that covered 52-genes. In this cohort, they identified five genetic alterations, including two KIT mutations, two NRAS and one MAPK2K1 mutation (38). Recently, Zhang et al. published a cohort of 17 NGGCT patients, where they were able to detect ctDNA in the CSF of 13 of the 17 patients at initial diagnosis. Importantly in this study, the investigators found that presence of ctDNA in the CSF after chemotherapy treatment to be prognostic. The NGS panel used to assess for ctDNA in this study covered 86 genes, and all CSF ctDNA found were copy number alterations in genes such as AKT2 and MAPK1, among others (39). These studies show proof-of-concept and the feasibility of evaluating CSF for ctDNA. However, larger cohorts (ideally with paired tissue) will be needed to determine the true frequency and reliability of capturing genomic alterations by ctDNA in CSF.

Recurrent chromosomal alterations, such as copy number gains, losses, and structural variants are the most common somatic alterations identified in GCTs. In a recent study, tumor analysis of intracranial GCTs showed that gain of 12p (a common alteration in testicular GCTs) is enriched in NGGCTs. Additionally, investigators from this study reported that the presence of 12p gain is associated with worse progression-free survival (PFS) and overall survival (OS), making this a potentially useful prognostic biomarker (40). Additionally, a gain of 3p25.3 has recently been reported as an independent poor prognostic factor for some extracranial and intracranial GCTs (41, 42). Given the potential prognostic value of these two chromosomal gains, one could consider ctDNA analysis for the presence of these alterations as a component for risk stratification.

Lastly, DNA methylation profiling is rapidly emerging as a valuable tool for the diagnosis of pediatric brain tumors. Currently, tissue samples have been utilized to create classifiers to diagnosis brain tumors, down to the level of genetic alteration subclassifications (43). Lack of robust intracranial GCT tissue samples representing all the various histology subtypes has made classifier challenging for this tumor type, but the German Cancer Research Center (DKZF) (https://www.molecularneuropathology.org) has incorporated some intracranial GCT histologic types, including germinoma, yolk sac and teratoma. Classification of the other NGGCT histologies has yet to be developed, and therefore the ability to classify mixed NGGCTs is still to be determined. Although further refinement is needed for intracranial GCT classification, differentiating germinoma from NGGCT can be distinguished by assessing the global DNA methylation patterns. Broadly, DNA methylation profiling of intracranial GCT tissue samples has shown that germinomas have global hypomethylation, while NGGCTs are globally hypermethylated (44).

As with other emerging molecular technologies, profiling intracranial GCTs has been hindered by the paucity of sufficient tissue samples for analysis. As the availability of tissue for patients can vary, the development of a liquid biopsy platform with ctDNA would be of great interest. Of note, the DKFZ methylation platform was developed based off the Illumina Infinium MethylationEPIC array platform, which calls for 250 ng of DNA input. The feasibility of obtaining 250 ng of ctDNA from CSF is unclear, as this would require large quantities of CSF. As such, other methylation sequencing platforms such as methylation DNA immunoprecipitation sequencing (MeDIP-seq) and enzymatic methyl sequencing (EM-seq) are being explored for DNA methylation profiling of lower inputs of DNA, such as cfDNA from CSF or plasma (45, 46). These technologies hold potential promise for developing cfDNA methylation profiling of CSF.

Taking all these emerging technologies and biomarkers into consideration, we are moving towards better means of diagnosing and stratifying IGCTs, which would be immensely helpful for treatment planning, risk stratification and in clinical trial design. These emerging technologies and biomarkers are summarized in **Table 1**.

**Table 1:** Emerging technologies and biomarkers.

Technique: Liquid Biopsy Platforms	Biomarker	Methods	Benefits/Uses	Limitations	Other Notes
Small-noncoding RNAs: microRNAs	microRNAs- miR-371-373, miR-302/367	qPCR, ddPCR	Highly sensitive and specific biomarker in extracranial GCTs	Larger numbers of CNS GCT samples need to be evaluated to validate sensitivity and specificity	miR-371a-3p most sensitive/specific in extracranial GCT
ctDNA: somatic mutations	KIT/KRAS/MAPK and AKT/mTOR alterations	NGS	CSF can be utilized to evaluate alterations in these pathways regardless of biopsy. Potentially identifying prognostic biomarkers (12p or 3p25.3 gain) or therapeutic targets.	Paired comparison of tissue and CSF needs to be performed to evaluate the frequency and reliability of capturing mutations.	NGS can capture point mutations, indels, CNVs, etc.
ctDNA: DNA methylation	Global DNA methylation, DKFZ Classifier	EM-seq, MeDIP-seq	Differential global methylation between germinoma vs. NGGCT can assist diagnosis without tissue biopsy.	DNA methylation classifier currently identifies germinoma, teratoma and yolk sac tumor but not other tumor types. Needs further validation in CSF samples.	Data can be used to evaluate for CNVs as well.

qPCR, quantitative (real-time) PCR; ddPCR, droplet digital PCR; CNS, central nervous system; GCT, germ cell tumor; ctDNA, circulating tumor DNA; NGS, next-generation sequencing; CSF, cerebrospinal fluid; CNV, copy number variation; DKFZ, German Cancer Research Center; EM-seq, enzymatic methyl sequencing; MeDIP-seq, methylation DNA immunoprecipitation sequencing; NGGCT, non-germinomatous germ cell tumor.

### Novel therapeutics and future trials

The advancement in our understanding of the molecular drivers of cancer has led to the development of biologic agents and targeted therapy for various malignancies. For intracranial GCTs, activating alterations in the MAPK pathway, including KIT, RAS, and PI3K/mTOR pathway, are known to be commonly seen in intracranial GCT (47, 48). KIT expression is of particular interest, as it is seen in the majority of pure germinoma and not seen among NGGCT without germinomatous component. In recent years, various inhibitors of KIT have been developed, with several gaining FDA approval for gastrointestinal stromal tumor (GIST) (49). With the success of targeted therapy in other pediatric indications, KIT inhibition has recently emerged as an intriguing potential treatment approach for CNS germinoma. Several trials have been proposed, both for recurrence disease as well as for upfront treatment (to potentially decrease the dose of chemotherapy needed for cure). These trials are actively under development.

Immunotherapy has also emerged as an effective treatment modality for a variety of cancers. Various immune checkpoint inhibitors have been approved for many malignancies, especially in adults. The role of immune checkpoint inhibitors in primary pediatric CNS malignancies, however, is unclear. One exception is for patients with constitutional mismatch repair deficiency syndrome (cMMRD) and high tumor mutational burden, where there is a clear indication and improved outcomes with immune checkpoint inhibition (50). In GCTs, there have been several case reports suggesting that this treatment modality may be of therapeutic potential in these tumors. This is evidenced by the durable responses reported in these cases with multiply recurrent/refractory disease (51–53). This includes a case of a multiply recurrent and refractory CNS NGGCT, who was treated with nivolumab and ipilimumab, resulting in a complete response and durable remission for over five years (51). Additionally, several recent studies have also demonstrated robust presence of tumor infiltrating lymphocytes and/or expression of immune checkpoint markers in both CNS germinoma and a subset of CNS NGGCT, further supporting the potential of this treatment modality in this patient population (54, 55).

For patients with recurrent CNS GCTs, trials with these innovative approaches are critically important to potentially expand therapeutic options and possibly augment the contemporary treatment paradigm, especially for recurrent disease. Additionally, if deemed effective, these treatments could be incorporated into the upfront treatment regimens, potentially decreasing the need for/dose of cytotoxic chemotherapy and radiation therapy, thereby reducing treatment related short- and long-term side effects.

## References

[1] Villano JL Propp JM Porter KR Stewart AK Valyi-Nagy T Li X. Malignant pineal germ-cell tumors: an analysis of cases from three tumor registries. Neuro Oncol. (2008) 10:121–30. doi: 10.1215/15228517-2007-054 PMC2613814 18287340

[2] Brown NJ. Special tumors of ovary and testis. Comparative pathology and histological identification. J Clin Pathol. (1977) 30:1089–. doi: 10.1136/jcp.30.11.1089-c

[3] Sano K Matsutani M Seto T. So-called intracranial germ cell tumours: personal experiences and a theory of their pathogenesis. Neurol Res. (1989) 11:118–26. doi: 10.1080/01616412.1989.11739874 2569683

[4] Echevarria ME Fangusaro J Goldman S. Pediatric central nervous system germ cell tumors: A review. Oncologist. (2008) 13:690–9. doi: 10.1634/theoncologist.2008-0037 18586924

[5] Murray MJ Bartels U Nishikawa R Fangusaro J Matsutani M Nicholson JC. Consensus on the management of intracranial germ-cell tumours. Lancet Oncol. (2015) 16:e470–e7. doi: 10.1016/S1470-2045(15)00244-2 26370356

[6] Allen J Chacko J Donahue B Dhall G Kretschmar C Jakacki R. Diagnostic sensitivity of serum and lumbar csf bhcg in newly diagnosed cns germinoma. Pediatr Blood Cancer. (2012) 59:1180–2. doi: 10.1002/pbc.24097 PMC3356788 22302772

[7] Hu M Guan H Lau CC Terashima K Jin Z Cui L. An update on the clinical diagnostic value of beta-hcg and alphafp for intracranial germ cell tumors. Eur J Med Res. (2016) 21:10. doi: 10.1186/s40001-016-0204-2 26968839 PMC4788851

[8] Fangusaro J Wu S MacDonald S Murphy E Shaw D Bartels U. Phase ii trial of response-based radiation therapy for patients with localized cns nongerminomatous germ cell tumors: A children’s oncology group study. J Clin Oncol. (2019) 37:3283–90. doi: 10.1200/JCO.19.00701 PMC6900864 31545689

[9] Calaminus G Frappaz D Kortmann RD Krefeld B Saran F Pietsch T. Outcome of patients with intracranial non-germinomatous germ cell tumors-lessons from the siop-cns-gct-96 trial. Neuro Oncol. (2017) 19:1661–72. doi: 10.1093/neuonc/nox122 PMC5716085 29048505

[10] Takami H Ichimura K. Biomarkers for risk-based treatment modifications for cns germ cell tumors: updates on biological underpinnings, clinical trials, and future directions. Front Oncol. (2022) 12:982608. doi: 10.3389/fonc.2022.982608 36132131 PMC9483213

[11] Pashankar F Murray MJ Gell J MacDonald N Shamash J Billmire DF. Consensus and controversy in the management of paediatric and adult patients with ovarian immature teratoma: the Malignant germ cell international consortium perspective. EClinicalMedicine. (2024) 69:102453. doi: 10.1016/j.eclinm.2024.102453 38544795 PMC10965411

[12] da Silva NS Cappellano AM Diez B Cavalheiro S Gardner S Wisoff J. Primary chemotherapy for intracranial germ cell tumors: results of the third international cns germ cell tumor study. Pediatr Blood Cancer. (2010) 54:377–83. doi: 10.1002/pbc.22381 20063410

[13] Kellie SJ Boyce H Dunkel IJ Diez B Rosenblum M Brualdi L. Intensive cisplatin and cyclophosphamide-based chemotherapy without radiotherapy for intracranial germinomas: failure of a primary chemotherapy approach. Pediatr Blood Cancer. (2004) 43:126–33. doi: 10.1002/pbc.20026 15236278

[14] Bamberg M Kortmann RD Calaminus G Becker G Meisner C Harms D. Radiation therapy for intracranial germinoma: results of the german cooperative prospective trials makei 83/86/89. J Clin Oncol. (1999) 17:2585–92. doi: 10.1200/JCO.1999.17.8.2585 10561326

[15] Calaminus G Kortmann R Worch J Nicholson JC Alapetite C Garre ML. Siop cns gct 96: final report of outcome of a prospective, multinational nonrandomized trial for children and adults with intracranial germinoma, comparing craniospinal irradiation alone with chemotherapy followed by focal primary site irradiation for patients with localized disease. Neuro Oncol. (2013) 15:788–96. doi: 10.1093/neuonc/not019 PMC3661100 23460321

[16] Bartels U Onar-Thomas A Patel SK Shaw D Fangusaro J Dhall G. Phase ii trial of response-based radiation therapy for patients with localized germinoma: A children’s oncology group study. Neuro Oncol. (2022) 24:974–83. doi: 10.1093/neuonc/noab270 PMC9159444 34850169

[17] Balmaceda C Heller G Rosenblum M Diez B Villablanca JG Kellie S. Chemotherapy without irradiation–a novel approach for newly diagnosed cns germ cell tumors: results of an international cooperative trial. The first international central nervous system germ cell tumor study. J Clin Oncol. (1996) 14:2908–15. doi: 10.1200/JCO.1996.14.11.2908 8918487

[18] Kellie SJ Boyce H Dunkel IJ Diez B Rosenblum M Brualdi L. Primary chemotherapy for intracranial nongerminomatous germ cell tumors: results of the second international cns germ cell study group protocol. J Clin Oncol. (2004) 22:846–53. doi: 10.1200/JCO.2004.07.006 14990640

[19] Dearnaley DP A’Hern RP Whittaker S Bloom HJ. Pineal and cns germ cell tumors: royal marsden hospital experience 1962-1987. Int J Radiat Oncol Biol Phys. (1990) 18:773–81. doi: 10.1016/0360-3016(90)90396-2 2323968

[20] Goldman S Bouffet E Fisher PG Allen JC Robertson PL Chuba PJ. Phase ii trial assessing the ability of neoadjuvant chemotherapy with or without second-look surgery to eliminate measurable disease for nongerminomatous germ cell tumors: A children’s oncology group study. J Clin Oncol. (2015) 33:2464–71. doi: 10.1200/JCO.2014.59.5132 PMC4507465 26101244

[21] Frappaz D Dhall G Murray MJ Goldman S Faure Conter C Allen J. Eano, sno and euracan consensus review on the current management and future development of intracranial germ cell tumors in adolescents and young adults. Neuro Oncol. (2022) 24:516–27. doi: 10.1093/neuonc/noab252 PMC8972311 34724065

[22] Aoyama H Shirato H Ikeda J Fujieda K Miyasaka K Sawamura Y. Induction chemotherapy followed by low-dose involved-field radiotherapy for intracranial germ cell tumors. J Clin Oncol. (2002) 20:857–65. doi: 10.1200/JCO.2002.20.3.857 11821471

[23] Shatara M Blue M Stanek J Liu YA Prevedello DM Giglio P. Final report of the phase ii next/cns-gct-4 trial: gempox followed by marrow-ablative chemotherapy for recurrent intracranial germ cell tumors. Neurooncol Pract. (2024) 11:188–98. doi: 10.1093/nop/npad067 PMC10940828 38496907

[24] Modak S Gardner S Dunkel IJ Balmaceda C Rosenblum MK Miller DC. Thiotepa-based high-dose chemotherapy with autologous stem-cell rescue in patients with recurrent or progressive cns germ cell tumors. J Clin Oncol. (2004) 22:1934–43. doi: 10.1200/JCO.2004.11.053 15143087

[25] Abu Arja MH Stanek JR Finlay JL AbdelBaki MS. Re-induction chemotherapy regimens in patients with recurrent central nervous system mixed Malignant germ cell tumors. Childs Nerv Syst. (2018) 34:2179–86. doi: 10.1007/s00381-018-3940-5 30076436

[26] Palmer RD Murray MJ Saini HK van Dongen S Abreu-Goodger C Muralidhar B. Malignant germ cell tumors display common microrna profiles resulting in global changes in expression of messenger rna targets. Cancer Res. (2010) 70:2911–23. doi: 10.1158/0008-5472.CAN-09-3301 PMC3000593 20332240

[27] Murray MJ Coleman N. Testicular cancer: A new generation of biomarkers for Malignant germ cell tumours. Nat Rev Urol. (2012) 9:298–300. doi: 10.1038/nrurol.2012.86 22549310

[28] Murray MJ Bell E Raby KL Rijlaarsdam MA Gillis AJ Looijenga LH. A pipeline to quantify serum and cerebrospinal fluid micrornas for diagnosis and detection of relapse in paediatric Malignant germ-cell tumours. Br J Cancer. (2016) 114:151–62. doi: 10.1038/bjc.2015.429 PMC4815809 26671749

[29] Lafin JT Kenigsberg AP Meng X Abe D Savelyeva A Singla N. Serum small rna sequencing and mir-375 assay do not identify the presence of pure teratoma at postchemotherapy retroperitoneal lymph node dissection. Eur Urol Open Sci. (2021) 26:83–7. doi: 10.1016/j.euros.2021.02.003 PMC8121258 33997822

[30] Dieckmann KP Radtke A Geczi L Matthies C Anheuser P Eckardt U. Serum levels of microrna-371a-3p (M371 test) as a new biomarker of testicular germ cell tumors: results of a prospective multicentric study. J Clin Oncol. (2019) 37:1412–23. doi: 10.1200/JCO.18.01480 PMC6544462 30875280

[31] Murray MJ Ajithkumar T Harris F Williams RM Jalloh I Cross J. Clinical utility of circulating mir-371a-3p for the management of patients with intracranial Malignant germ cell tumors. Neurooncol Adv. (2020) 2:vdaa048. doi: 10.1093/noajnl/vdaa048 32642701 PMC7236383

[32] Schonberger S Mohseni MM Ellinger J Tran GVQ Becker M Claviez A. Microrna-profiling of mir-371~373- and mir-302/367-clusters in serum and cerebrospinal fluid identify patients with intracranial germ cell tumors. J Cancer Res Clin Oncol. (2023) 149:791–802. doi: 10.1007/s00432-022-03915-4 35171328 PMC9931786

[33] Phi JH Kim SK Lee YA Shin CH Cheon JE Kim IO. Latency of intracranial germ cell tumors and diagnosis delay. Childs Nerv Syst. (2013) 29:1871–81. doi: 10.1007/s00381-013-2164-y 23811803

[34] Tong T Chen H Mo C Zhong L. Clinical characteristics and predictive factors of delayed diagnosis in patients with sellar germ cell tumors. J Neurooncol. (2024) 167:467–76. doi: 10.1007/s11060-024-04626-1 38438767

[35] Sun Y Li M Ren S Liu Y Zhang J Li S. Exploring genetic alterations in circulating tumor DNA from cerebrospinal fluid of pediatric medulloblastoma. Sci Rep. (2021) 11:5638. doi: 10.1038/s41598-021-85178-6 33707557 PMC7952732

[36] Chicard M Iddir Y Masliah Planchon J Combaret V Attignon V Saint-Charles A. Cell-free DNA extracted from csf for the molecular diagnosis of pediatric embryonal brain tumors. Cancers (Basel). (2023) 15(13):3532. doi: 10.3390/cancers15133532 37444642 PMC10340330

[37] Liu APY Smith KS Kumar R Robinson GW Northcott PA. Low-coverage whole-genome sequencing of cerebrospinal-fluid-derived cell-free DNA in brain tumor patients. STAR Protoc. (2022) 3:101292. doi: 10.1016/j.xpro.2022.101292 35463474 PMC9026582

[38] Takayasu T Shah M Dono A Yan Y Borkar R Putluri N. Cerebrospinal fluid ctdna and metabolites are informative biomarkers for the evaluation of cns germ cell tumors. Sci Rep. (2020) 10:14326. doi: 10.1038/s41598-020-71161-0 32868820 PMC7459305

[39] Zhang YT Jin XM Zhong XD Chang J. Monitoring pediatric cns non-germinomatous germ cell tumors *via* cerebrospinal fluid circulating tumor DNA. Pediatr Blood Cancer. (2024) 71(11):e31288. doi: 10.1002/pbc.31288 39189644

[40] Satomi K Takami H Fukushima S Yamashita S Matsushita Y Nakazato Y. 12p gain is predominantly observed in non-germinomatous germ cell tumors and identifies an unfavorable subgroup of central nervous system germ cell tumors. Neuro Oncol. (2022) 24:834–46. doi: 10.1093/neuonc/noab246 PMC9071297 34698864

[41] Takami H Satomi K Fukuoka K Nakamura T Tanaka S Mukasa A. Distinct patterns of copy number alterations may predict poor outcome in central nervous system germ cell tumors. Sci Rep. (2023) 13:15760. doi: 10.1038/s41598-023-42842-3 37735187 PMC10514291

[42] Timmerman DM Eleveld TF Sriram S Dorssers LCJ Gillis AJM Schmidtova S. Chromosome 3p25.3 gain is associated with cisplatin resistance and is an independent predictor of poor outcome in male Malignant germ cell tumors. J Clin Oncol. (2022) 40:3077–87. doi: 10.1200/JCO.21.02809 PMC9462533 35442716

[43] Capper D Stichel D Sahm F Jones DTW Schrimpf D Sill M. Practical implementation of DNA methylation and copy-number-based cns tumor diagnostics: the heidelberg experience. Acta Neuropathol. (2018) 136:181–210. doi: 10.1007/s00401-018-1879-y 29967940 PMC6060790

[44] Fukushima S Yamashita S Kobayashi H Takami H Fukuoka K Nakamura T. Genome-wide methylation profiles in primary intracranial germ cell tumors indicate a primordial germ cell origin for germinomas. Acta Neuropathol. (2017) 133:445–62. doi: 10.1007/s00401-017-1673-2 28078450

[45] Landry AP Zuccato JA Patil V Voisin MR Wang JZ Ellenbogen Y. Establishing the utility of multi-platform liquid biopsy by integrating the csf methylome and proteome in cns tumours. J Neurooncol. (2024) 169:233–9. doi: 10.1007/s11060-024-04695-2 PMC11915530 39102117

[46] Trinidad EM Vidal E Coronado E Esteve-Codina A Castel V Canete A. Liquidhope: methylome and genomic profiling from very limited quantities of plasma-derived DNA. Brief Bioinform. (2023) 24. doi: 10.1093/bib/bbac575 PMC9851319 36611239

[47] Takami H Fukuoka K Fukushima S Nakamura T Mukasa A Saito N. Integrated clinical, histopathological, and molecular data analysis of 190 central nervous system germ cell tumors from the igct consortium. Neuro Oncol. (2019) 21:1565–77. doi: 10.1093/neuonc/noz139 PMC6917411 31420671

[48] Wang L Yamaguchi S Burstein MD Terashima K Chang K Ng HK. Novel somatic and germline mutations in intracranial germ cell tumours. Nature. (2014) 511:241–5. doi: 10.1038/nature13296 PMC4532372 24896186

[49] Bauer S George S von Mehren M Heinrich MC. Early and next-generation kit/pdgfra kinase inhibitors and the future of treatment for advanced gastrointestinal stromal tumor. Front Oncol. (2021) 11:672500. doi: 10.3389/fonc.2021.672500 34322383 PMC8313277

[50] Das A Tabori U Sambira Nahum LC Collins NB Deyell R Dvir R. Efficacy of nivolumab in pediatric cancers with high mutation burden and mismatch repair deficiency. Clin Cancer Res. (2023) 29:4770–83. doi: 10.1158/1078-0432.CCR-23-0411 PMC10690097 37126021

[51] Cacciotti C Choi J Alexandrescu S Zimmerman MA Cooney TM Chordas C. Immune checkpoint inhibition for pediatric patients with recurrent/refractory cns tumors: A single institution experience. J Neurooncol. (2020) 149:113–22. doi: 10.1007/s11060-020-03578-6 32627129

[52] Li B Zhao S Li S Li C Liu W Li L. Novel molecular subtypes of intracranial germ cell tumors expand therapeutic opportunities. Neuro Oncol. (2024) 26:1335–51. doi: 10.1093/neuonc/noae038 PMC11226877 38430549

[53] Zschabitz S Lasitschka F Hadaschik B Hofheinz RD Jentsch-Ullrich K Gruner M. Response to anti-programmed cell death protein-1 antibodies in men treated for platinum refractory germ cell cancer relapsed after high-dose chemotherapy and stem cell transplantation. Eur J Cancer. (2017) 76:1–7. doi: 10.1016/j.ejca.2017.01.033 28262583

[54] Woods JK Lidov HG Ligon KL Santagata S Chi SN Yeo KK. Pd-L1 and pd-1 expression in pediatric central nervous system germ cell tumors. Mod Pathol. (2022) 35(12):1770–4. doi: 10.1038/s41379-022-01142-3 36057740

[55] Liu B Arakawa Y Yokogawa R Tokunaga S Terada Y Murata D. Pd-1/pd-L1 expression in a series of intracranial germinoma and its association with foxp3+ and cd8+ Infiltrating lymphocytes. PloS One. (2018) 13:e0194594. doi: 10.1371/journal.pone.0194594 29617441 PMC5884516

